# Safety of oral afoxolaner formulated with or without milbemycin oxime in homozygous MDR1‐deficient collie dogs

**DOI:** 10.1111/jvp.13064

**Published:** 2022-05-10

**Authors:** Marlene Drag, Eric Tielemans, Elizabeth Mitchell

**Affiliations:** ^1^ Boehringer Ingelheim Animal Health, Missouri Research Center Fulton Missouri USA; ^2^ Boehringer Ingelheim Animal Health Lyon France; ^3^ Boehringer Ingelheim Animal Health Athens Georgia USA

**Keywords:** afoxolaner, macrocyclic lactone, MDR1 deficient dog, milbemycin oxime, safety

## Abstract

Afoxolaner, an insecticide and acaricide compound of the isoxazoline class, is available for dogs as an oral ectoparasiticide medicine (NexGard®) and as an oral endectoparasiticide medicine in combination with milbemycin oxime (MO), a macrocyclic lactone (NexGard® Spectra). The safety of these two compounds, alone or in combination, was investigated in homozygous MDR1‐deficient collie dogs, in two studies. Overall, 30 adult collie dogs were treated once orally, 9 with a placebo, 9 with afoxolaner, 6 with MO, and 6 with a combination of afoxolaner and MO. For afoxolaner, the mean investigated dosage corresponded to 3.8 and 4.7 multiples of the maximum recommended therapeutic doses (RTD) in NexGard® and NexGard® Spectra, respectively. For MO, the mean investigated dosage corresponded to 4.7 multiples of the maximum RTD in NexGard® Spectra. Dogs were closely monitored for adverse reactions on the day of treatment and for the following two days. No significant adverse reaction was observed in any dog from the afoxolaner or the afoxolaner + MO groups; in the MO‐only treated group, mild and transient neurological signs were observed during the 4–8 h post‐treatment window. These studies demonstrated a high level of safety of oral afoxolaner, alone or in combination with milbemycin oxime, in homozygous MDR1‐deficient dogs.

## 1 INTRODUCTION

P‐glycoprotein is a well‐characterized mammalian plasma membrane efflux pump, located in brain capillary endothelial cells and several other tissues such as intestinal mucosae, kidney, and liver (Borst & Schinkel, 2013; Mealey, 2008; Schinkel et al., 1995). It is an essential component of the blood–brain barrier for the protection of mammalian species' neurones from circulating xenobiotic molecules. P‐glycoprotein also impacts the pharmacokinetic properties of orally administered molecules by impeding intestinal uptake and increase kidney and liver clearance of circulating molecules (Mealey, 2008; Schinkel, 1997; Schinkel et al. 1995). P‐glycoprotein substrates are large hydrophobic molecules and include several families of compounds, such as opioids, cyclosporins, digoxin, dexamethasone, and macrocyclic lactones (Kwei et al., 1999; Schinkel et al., 1995). P‐glycoprotein is coded by the ABCB1 gene, formerly known as the multi‐drug resistance gene, MDR1 (in this manuscript either “ABCB1” or “MDR1” terms are used to describe this gene). An ABCB1 mutation with consequent P‐glycoprotein deficiency is widely distributed in the collie dog population, 35% of the dogs were homozygous and more than 75% had at least one mutant allele (Mealey & Meurs, 2008; Neff et al., 2004). This deficiency also affects other herding type breeds, such as Australian shepherds, German shepherds, Windsprites, and old English sheepdogs (Mealey et al., 2001; Monobe et al., 2015; Neff et al., 2004; Nelson et al., 2003), or related crossbreeds (Dekel et al., 2017). Dogs homozygous for the ABCB1 defect have the potential to have dose‐dependent reactions to P‐glycoprotein substrates. Dogs which are heterozygous for the ABCB1 defect may be more sensitive to P‐glycoprotein substrates than normal (wild type) dogs (Dowling, 2006) but to a lesser consistency (Sherman et al., 2010). The ABCB1 genetic deficiency has also been described in cats suffering from neurological signs following macrocyclic lactone exposure (Mealey et al., 2021; Mealey & Burke, 2015).

Cys‐loop receptors, highly conserved ligand‐gated ion channels of vertebrates, arthropods, and nematodes, are located in neurons of peripheral and central nervous systems. They mediate synaptic neurotransmissions and include γ‐aminobutyric acid‐ (GABA) gated chloride channels, glutamate‐gated chloride channels (GluCl), serotonine type 3 (5HT_3_) receptors, nicotinic acetylcholine (nACh), and glycine (Gly) receptors (Sparling & DiMauro, 2017; Xue et al., 2021). Many parasiticide drugs target Cys‐loop receptors, especially GABA‐ and glutamate‐gated chloride channels, both substrates being inhibitory neurotransmitters (Ozoe, 2013).

The isoxazoline parasiticide molecules antagonize GABA receptors of arthropods (Ozoe et al., 2010; Selzer & Epe, 2021; Shoop et al., 2014; Weber & Selzer, 2016). Macrocyclic lactones (ML) activate GluCl channel receptors on nerves and muscular cells of arthropods and nematodes (Noack et al., 2021; Yates & Wolstenholme, 2004). Consequently, both isoxazolines and MLs activate electrophysiological disruption of the nervous system, causing death of the parasite. Glutamate‐gated chloride channels are absent from vertebrates (Merola & Eubig, 2012; Noack et al., 2021; Wolstenholme, 2012), and thus there is no potential for neurotoxicity in mammals in relation to ML/GluCl interaction. However, MLs also have affinity for GABA‐gated chloride channels. GABA receptors, namely the vertebrate sub‐type GABA_A_, are prominent and similar in nematodes, arthropods, and vertebrates and therefore MLs have a potential for neurotoxicity in mammalian species (Merola & Eubig, 2012; Sieghart, 2006; Sigel & Steinmann, 2012; Simeone et al., 2003; Soualah et al., 2021). MLs have been extensively used since the early 1980s as parasiticides in veterinary and human medicine (Crump & Ōmura, 2011; Nolan & Lok, 2012).

The blood–brain barrier protects mammals' brains from isoxazolines and MLs; however, fractions of MLs may permeate the blood–brain barrier with potential neurological reactions (Merola & Eubig, 2012; Trailovic & Nedeljkovic, 2011). This is especially true in P‐glycoprotein‐deficient dogs, which may suffer from brain accumulation of MLs, recognized P‐glycoprotein substrates, in their central nervous system, with subsequent neurotoxic signs such as ataxia, tremors, seizure, mydriasis, lethargy, or coma (Mealey et al., 2001; Paul et al., 2000). Nevertheless, the label doses of MLs in canine parasiticide medicines have been demonstrated safe in MDR1‐deficient dogs (Geyer & Janko, 2012; Paul et al., 2000; Sherman et al., 2010; Tranquilli et al., 1991). Beside a clear dose dependency, the safety of MLs in dogs (and to a much higher extent in MDR1‐deficient dogs) is also dependent on the route of administration. As an illustration, the moxidectin dose of Simparica® Trio, an oral canine endectoparasiticide product is 24–48 μg/kg. In a study using MDR1‐deficient dogs, an acceptable margin of safety was demonstrated for this product at 1x and 3x multiples of the maximum recommended dose of 48 μg/kg; however, some signs of ML toxicity were observed at 5x (EMA, 2021). In the topical Advantage® Multi/Advocate®, moxidectin was applied at up to 260x multiples of the oral dose of Simparica® Trio, at 2.5–6.25 mg/kg and was demonstrated safe in ivermectin‐sensitive collie dogs (EMA, 2009; Paul et al., 2004).

Afoxolaner, an isoxazoline compound, has been used for several years in dogs as an oral ectoparasiticide medicine (NexGard®) (EMA, 2013b; FDA, 2013; Selzer & Epe, 2021; Weber & Selzer, 2016) and as an oral endectoparasiticide medicine, in combination with milbemycin oxime (NexGard® Spectra) (EMA, 2014). Milbemycin oxime (MO) belongs to the ML family of molecules (Merola & Eubig, 2012; Noack et al., 2021; Vercruysse & Rew, 2002). Both afoxolaner and afoxolaner combined with MO have been demonstrated safe in beagle dogs at up to 5 times the maximum exposure dose (Drag et al., 2014; Drag et al., 2017).

As the penetration of MLs into the brain and their effect on GABA_A_ receptors may be increased in P‐glycoprotein‐deficient dogs, and as the relevance of afoxolaner to P‐glycoprotein is unknown, it is important to verify the safety of afoxolaner, alone and especially in combination with MO, in these dogs. This manuscript describes two studies designed for the testing of the margin for the safety of afoxolaner and afoxolaner combined with milbemycin oxime in MDR1‐defective dogs.

## 2 MATERIALS AND METHODS

The studies were conducted with healthy purpose‐bred homozygous MDR1‐deficient collie dogs, aged 2.2–10.9 years and weighing 21–41 kg. The numbers, sex, average age, and bodyweight of dogs in each study are described in Table 1. Blood samples had previously been genotyped to confirm that the dogs were homozygous for the MDR1 mutation. All dogs had previously demonstrated sensitivity to ivermectin administered orally at 100–120 μg/kg. Signs of sensitivity were evaluated using a specific evaluation scoring system of ML toxicity signs (Table 2). All collies had mild changes in at least two of the following parameters: coordination/gait, mental status/depression, or salivation. The studies used a blinded, negative (placebo) controlled and randomized design. In the first study, dogs were randomized on the basis of ascending numerical identification, and in the second study they were randomized on the basis of age.

**TABLE 1:** Study groups, animals, and dosages

	Group	Number of male (M) and female (F) MDR1 deficient Colley dogs	Average body weight (kg)	Average age (years)	Average dosage (mg/kg)	
					Afoxolaner	MO
Study 1	Placebo	3 F	29.83	4.0	NA	NA
	Afoxolaner	4 M, 5F	25.12	5.7	26.7 (3.8)^a^	NA
Study 2	Placebo	5 M, 1F	31.37	6.5	NA	NA
	MO	4 M, 2F	33.53	6.5	NA	5.2
	Afoxolaner + MO	4 M, 2F	34.65	6.2	25.6 (4.7)^b^	5.1 (4.7)^b^

^a^ multiple of maximum label dose in NexGard™.

^b^ multiple of maximum label dose in NexGard™ Spectra.

**TABLE 2:** Description of signs (nature and level) attributed to macrocyclic lactone neurological reaction

Pupillary light reflex	Normal	Typical brisk pupil closure, both direct and indirect pupil reflexes intact.
	Mild	Pupil closure delayed, both direct and indirect pupil reflexes intact.
	Moderate	Pupil closure delayed and indirect pupil reflex absent.
	Marked	Pupils remain dilated.
Mental status/Depression	Normal	Normal response to stimuli.
	Mild	Lethargy, response to stimuli somewhat depressed. Appears confused.
	Moderate	Semi‐responsive. Will only respond to persistent stimulus.
	Marked	Recumbent, weak, non‐responsive. Little or no response to stimulus, including deep pain (paw pinch). Unaware of surroundings.
Coordination/Gait	Normal	Usual gait and posture.
	Mild	Detectable incoordination or tremors, but moves without much difficulty and able to stand on own.
	Moderate	Able to stand on own but moves with difficulty due to incoordination/ataxia.
	Marked	Unable to remain standing without support or unable to rise for recumbency due to incoordination ataxia.
Salivation	Normal	Thin and watery (dry or wet mouth).
	Mild	Increased volume and thickness.
	Moderate	Persistent thick salivation.
	Marked	Pooling persistent thick salivation

The first study investigated the safety of afoxolaner; the second study investigated the safety of MO, alone or in combination with afoxolaner. The dogs were treated once orally with experimental chewable formulations administered in numbers to provide a dose as close as possible to 25 mg/kg afoxolaner, and/or 5 mg/kg MO, that is, approximately 10 times the minimum recommended therapeutic dose (RTD) dose (2.7 mg/kg afoxolaner in NexGard®, 2.5 and 0.5 mg/kg afoxolaner and MO respectively in NexGard® Spectra) (Table 1). In Study 1, the mean afoxolaner dose of 26.7 mg/kg corresponded to 9.9 and 3.8 multiples of the minimum and maximum RTD of Nexgard® (i.e., 2.7 and 7.1 mg/kg) respectively. In Study 2, the mean dose of 25.6 mg/kg afoxolaner corresponded to 10.2 and 4.7 multiples of the minimum and maximum RTD of Nexgard® Spectra (i.e., 2.5 and 5.4 mg/kg), respectively; and the mean dose of 5.2 mg/kg MO corresponded to 10.2 and 4.7 multiples of the minimum and maximum RTD of Nexgard® Spectra (i.e., 0.5 and 1.1 mg/kg), respectively. The MO‐only group received a mean dose of 5.2 mg/kg. In both studies, dogs were treated after feeding.

The personnel masked to treatment observed the dogs for adverse reactions before treatment then 2, 4, 6, 8, and 12 h after treatment and twice a day (am and pm) for the next 2 days. Beside general health, clinical observations including a specific evaluation scoring system of ML toxicity signs are detailed in Table 2.

## 3 RESULTS

In Study 1, no neurological reaction or significant treatment‐related adverse reaction was observed. In the afoxolaner group, two adverse reactions with unclear relation to the treatment were observed, one dog vomited 6 h, and another dog had diarrhea 8 h after the afoxolaner dose.

In Study 2, one instance of vomiting was observed, 2 h after the MO‐only treatment, with unclear relationship to the treatment. Neurological signs corresponding to ML‐related reactions were observed in the three groups and are detailed in Table 3. All signs were scored as mild or “normal‐mild.” The “normal‐mild” score was used when the observers deemed the intensity/consistency of a sign below the provided description for a mild score. Most signs had little clinical significance, by their duration and intensity and none required veterinary care. Almost half (9 in 20) of the pupillary light reflex changes were observed before treatment or in the placebo group, demonstrating that such observations can be subjective and have no clinical significance, and none of the pre‐dose observations raised any concern for inclusion in the study. Salivations were mostly rated “normal‐mild,” and some also occurred in the placebo group. Therefore when “pupillary light reflex” changes and/or “salivation” were the only signs observed, the results were considered unlikely to indicate ML toxicity, which was applicable to 2 dogs of the MO‐only group, and to 5 dogs of the MO + afoxolaner group. Normal‐mild or mild signs of mental status/depression and/or coordination/gait were observed in three of the 6 dogs in the MO‐only group. Trembling was also described for one of the three dogs. Changes in mental status/depression and/or coordination/gait are more definitive of ML toxicity than variable changes in salivation and pupil dilation because these signs more accurately reflect the presence of MLs in the CNS. Mental and gait changes are normally present at more than one consecutive timepoint because eliminations of MLs also appear to be affected by the lack of P‐glycoprotein. The results attributed to the ML treatment confirmed the sensitivity of MDR1‐deficient dogs to elevated doses (4.7 multiples of the maximum label doses) of MO. The results also demonstrate that reactions among MDR1‐deficient dogs administered MLs at levels lower than severely toxic doses can be variable.

**TABLE 3:** Neurological signs scorings in study 2

Dog #^a^	Group^b^	Nature^c^	Severity^c^	Timing of observation(s)^d^	Other observation
Dog 1	Placebo	Pupillary light reflex	Mild	pre‐dose, 2 h	
Dog 2	Placebo	Pupillary light reflex	Mild	pre‐dose	
		Salivation	Normal‐Mild^e^	6 h, 8 h	
Dog 3	MO	Pupillary light reflex	Mild	4 h, 12 h	Trembling at 4 h
		Salivation	Normal‐Mild^e^	4 h, 8 h, 12 h, Day 1 am	
		Salivation	Mild	6 h	
		Mental status/Depression	Normal‐Mild^e^	4 h, 8 h, 12 h	
		Mental status/Depression	Mild	6 h	
		Coordination/Gait	Normal‐Mild^e^	8 h, 12 h	
		Coordination/Gait	Mild	4 h, 6 h	
Dog 4	MO	Salivation	Normal‐Mild^e^	6 h, 8 h	
Dog 5	MO	Salivation	Normal‐Mild^e^	6 h	
		Mental status/Depression	Normal‐Mild^e^	6 h, 8 h, 12 h	
		Coordination/Gait	Normal‐Mild^e^	6 h	
Dog 6	MO	Pupillary light reflex	Mild	pre‐dose, 2 h, 4 h, 12 h	
		Salivation	Normal‐Mild^e^	8 h	
Dog 7	MO	Salivation	Normal‐Mild^e^	4 h, 12 h, Day 1 am	
		Salivation	Mild	6 h, 8 h	
		Coordination/Gait	Normal‐Mild^e^	6 h	
		Coordination/Gait	Mild	8 h	
Dog 8	MO + Afoxolaner	Pupillary light reflex	Mild	pre‐dose	
Dog 9	MO + Afoxolaner	Pupillary light reflex	Mild	pre‐dose, 2 h, 4 h, 12 h, Day 2 am	
Dog 10	MO + Afoxolaner	Pupillary light reflex	Mild	2 h	
Dog 11	MO + Afoxolaner	Pupillary light reflex	Mild	pre‐dose	
Dog 12	MO + Afoxolaner	Pupillary light reflex	Mild	pre‐dose	
		Salivation	Normal‐Mild^e^	8 h	
Dog 13	MO + Afoxolaner	Pupillary light reflex	Normal‐Mild^e^	pre‐dose, Day 2 am	

^a^ Only inclusive of dogs observed with signs other than “normal” as described in Table 2.

^b^ There were 6 dogs in each, the placebo, the milbemycin oxime (MO), and the afoxolaner + milbemycin oxime (MO + Afoxolaner) groups.

^c^ See Table 2 for descriptions.

^d^ Each dog was examined on Day 0, pre‐dose then 2 h, 4 h, 6 h, 8 h, 12 h, on Day 1 am, Day 1 pm, Day 2 am, and Day 2 pm.

^e^ signs were scored as normal‐mild, when the observers deemed the intensity/consistency of a sign below the provided description for a mild level as described in Table 2.

## 4 DISCUSSION

The two studies described in this manuscript provide the first in vivo investigations about afoxolaner safety in MDR1 mutant dogs. Recognized P‐glycoprotein substrates are generally amphiphilic hydrophobic xenobiotic molecules of natural or semisynthetic origin (Kwei et al., 1999; Schinkel et al., 1995). Little is known about isoxazoline interactions with P‐glycoprotein. A study using knock out MDR1 mice has demonstrated that the brain penetration of fluralaner, another isoxazoline compound, is partially regulated by MDR1 P‐glycoproteins, although to a much lower extent than ivermectin (Geyer et al., 2018). It is difficult to extrapolate observations prepared on genetically modified mice to MDR1‐deficient dogs, and to hypothesize a clinical significance of the observed partial P‐glycoprotein regulation.

Study 1 demonstrated a high level of safety of afoxolaner administered once in MDR1‐deficient collie dogs, on average at a 3.8 multiple (26.7 mg/kg) of the maximum recommended therapeutic dose (RTD) in NexGard®. These results were in line with other canine isoxazoline products, for example, fluralaner (EMA, 2013a; Walther et al., 2014) or sarolaner (EMA, 2015b) for which safety in MDR1‐deficient collie dogs was demonstrated at three times the RTD. Study 1 confirmed that afoxolaner, a small, lipophilic and unionized compound that easily crosses cell membranes via passive diffusion (Letendre et al., 2014) was well tolerated by dogs with MDR1 P‐glycoprotein blood–brain barrier deficiencies.

Study 2 demonstrated safety in MDR1‐deficient collie dogs of afoxolaner (25.6 mg/kg) combined with MO (5.1 mg/kg), administered at 4.7 multiples of the (maximum) RTD in NexGard™ Spectra. These results were partly in line with Credelio® Plus, an oral endectoparasiticide product for dogs combining the isoxazoline lotilaner with MO (EMA, 2015a). The safety of Credelio® Plus in MDR1 deficient collie dogs was demonstrated at 2.9‐fold RTD but was not demonstrated at 4.8‐fold RTD, per cumulative avermectin sensitivity scores exceeding the scores of the lower dose (1 and 2.9‐fold RTD) and placebo groups (EMA, 2015b). As the RTD of MO in Credelio® Plus (0.75–1.53 mg/kg) is 50% higher than in NexGard™ Spectra (0.5–1.07 mg/kg), the intermediate dose in the Credelio study (2.9 fold maximum RTD, 4.4 mg/kg MO) which was proven safe, is more comparable to the 4.7‐fold RTD (5.1 mg/kg MO) dose in NexGard® Spectra in Study 2. Nevertheless, even though NexGard® Spectra has a lower MO dose than Credelio® Plus, it is important to strictly follow the dosage instructions of the product to ensure safety of any dog with P‐glycoprotein deficiency. Study 2 also allowed the verification that MO administered orally alone at 5.2 mg/kg on average, triggered some mild and self‐resolving ML‐related neurological reactions. This was in line with previous scientific reports, for example, in a dose escalating study, the “no side‐effect dose” of oral MO in collie dogs was determined at 1.25 mg/kg (Bishop et al., 2000); in another study, mild depression was observed in some collie dogs on the day of a treatment with 5 mg/kg MO and more ML‐related neurological clinical signs (depression, ataxia, mydriasis, and salivation) were observed in all collie dogs treated with 10 mg/kg (Tranquilli et al., 1991). Furthermore, the MO‐only treated group in Study 2 allowed the confirmation that the tested collie dogs were sensitive to MLs through the observations of ML‐related neurological adverse reactions.

In Study 2, all significant ML‐related neurological reactions were observed in the MO‐only treated group and none in the MO + afoxolaner treated group despite identical MO dosages. Both MO and afoxolaner have a pharmacodynamic effect at the level of GABA_A_ receptors. The GABA receptor level site of afoxolaner is different from the GABA receptor site of MLs on the ion channel (Noack et al., 2021; Shoop et al., 2014; Weber & Selzer, 2016), where afoxolaner has an antagonist effect while MO an agonist effect. It is then possible that both compounds interact at the GABA_A_ level with consequent decreased level of electrophysiological disruptions in the synaptic ion channel. More specific research may support this hypothesis.

In summary, these two studies demonstrated a high level of safety of afoxolaner (NexGard®) and oral combination of afoxolaner and milbemycin oxime (NexGard® Spectra) administered orally in MDR1‐deficient dogs.

## CONFLICT OF INTEREST

The work reported herein was funded by Boehringer Ingelheim. The authors are the current employees of Boehringer Ingelheim Animal Health. This document is provided for scientific purposes only. Any reference to a brand or trademark herein is for information purposes only and is not intended for any commercial purposes or to dilute the rights of the respective owners of the brand(s) or trademark(s). NexGard® and NexGard® Spectra are registered trademarks of Boehringer Ingelheim Animal Health.

## AUTHOR CONTRIBUTIONS

Marlene Drag and Elizabeth Mitchell designed, monitored, and supervised the studies. Eric Tielemans drafted the manuscript. All authors contributed to and approved the final version of the manuscript.

## ANIMAL WELFARE

Animals were handled similarly and with due regard for their well‐being.

## Data Availability

The data from these studies are not publicly available because they contain proprietary information of registered veterinary products.
